# Application of DNA Quadruplex Hydrogels Prepared from Polyethylene Glycol-Oligodeoxynucleotide Conjugates to Cell Culture Media

**DOI:** 10.3390/polym11101607

**Published:** 2019-10-02

**Authors:** Shizuma Tanaka, Shinsuke Yukami, Yuhei Hachiro, Yuichi Ohya, Akinori Kuzuya

**Affiliations:** 1 Department of Chemistry and Materials Engineering, Kansai University, 3-3-35 Yamate, Suita, Osaka 564-8680, Japan; k170625@kansai-u.ac.jp (S.T.); k507837@kansai-u.ac.jp (S.Y.); k962541@kansai-u.ac.jp (Y.H.); 2 Collaborative Research Center of Engineering, Medicine, and Pharmacology, ORDIST, Kansai University, 3-3-35 Yamate, Suita, Osaka 564-8680, Japan

**Keywords:** hydrogels, DNA, G-quadruplexes, polyethylene glycol, cell culture

## Abstract

Application of Na^+^-responsive DNA quadruplex hydrogels, which utilize G-quadruplexes as crosslinking points of poly(ethylene glycol) (PEG) network as cell culture substrate, has been examined. PEG-oligodeoxynucleotide (ODN) conjugate, in which four deoxyguanosine (dG4) residues are tethered to both ends of PEG, was prepared by modified high-efficiency liquid phase (HELP) synthesis of oligonucleotides and used as the macromonomer. When mixed with equal volume of cell culture media, the solution of PEG-ODN turned into stiff hydrogel (G-quadruplex hydrogel) as the result of G-quadruplex formation by the dG4 segments in the presence of Na^+^. PEG-ODN itself did not show cytotoxicity and the resulting hydrogel was stable enough under cell culture conditions. However, L929 fibroblast cells cultured in G-quadruplex hydrogel remained spherical for a week, yet alive, without proliferation. The cells gradually sedimented through the gel day by day, probably due to the reversible nature of G-quadruplex formation and the resulting slow rearrangement of the macromonomers. Once they reached the bottom glass surface, the cells started to spread and proliferate.

## 1. Introduction

Recent development of various hydrogels with unique properties is attracting scientists not only from material chemistry, but also from broad research areas such as medical science to the field [1,2]. DNA is one of the well-studied components used to prepare hydrogels [3,4,5,6,7,8,9,10,11], because of its sophisticated functions as a fruit of previous studies in DNA computing and structural DNA nanotechnology [12,13]. Not only regular duplexes [4], but also four-stranded structures called “G-quadruplex” and “i-motif” are often used to construct DNA hydrogels due to their attractive features: metal ion or pH responsiveness [14,15,16,17,18,19,20,21,22,23,24]. We have recently developed a new class of hydrogels utilizing such DNA quadruplexes as cross-linking points of the 3D polymer network [25,26,27]. Polyethylene glycol-oligodeoxynucleotide (PEG-ODN) conjugates bearing merely four deoxyguanosine residues (dG4) for G-quadruplexes or five deoxycytidine residues (dC5) for i-motifs at the ends of linear or four-way branched PEG, were prepared as macromonomers by applying high-efficiency liquid phase (HELP) synthesis of oligonucleotides developed by Bonora et al. with a few modifications [28]. This technique enabled us to prepare typically 10−20 g of PEG-ODN conjugates in laboratories and to overcome the size-barrier of popular solid-phase DNA synthesis [29], which only produces oligonucleotides in less than milligrams. PEG-dG4 conjugates prepared as such gave intelligent, biodegradable, and self-healing hydrogels (G-quadruplex hydrogels), which turned into hydrogel quite rapidly upon addition of various body-related fluids containing Na^+^, such as serum, artificial sweat or tear, or even simple phosphate buffered saline (PBS). Na^+^-responsive G-quadruplex hydrogels, thus, have been considered as a potentially useful biomaterial for widespread biomedical applications.

**Figure 1:**
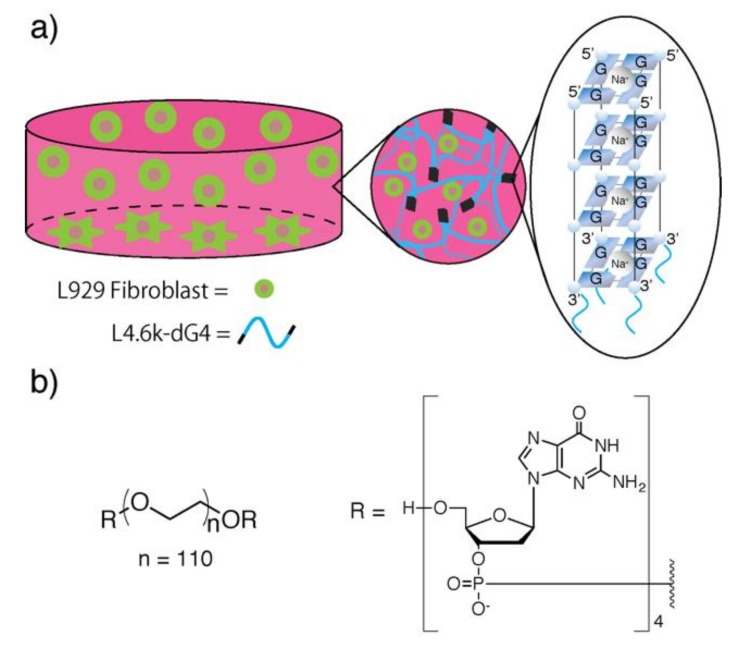
(**a**) Schematic illustration of cell culture on/in G-quadruplex hydrogels. Wavy blue lines represent poly(ethylene glycol) (PEG) segments. Upon the addition of cell culture media to L4.6k-dG4 conjugate solution, tetradeoxyguanosine (dG4) segments immediately form G-quadruplexes in response to Na^+^, to crosslink PEG segments into a 3D network. L929 fibroblast cells, either dispersed in the hydrogel or adhered to the bottom of the well, are presented in green circles or stars, respectively. (**b**) Chemical structure of L4.6k-dG4.

In this study, another possible application of G-quadruplex hydrogel as cell culture scaffold has been studied (Figure 1). Hydrogel formation in response to Na^+^ in typical cell culture media was first examined. After the stability of the resulting G-quadruplex hydrogels under cell culture conditions was confirmed, the hydrogels were treated together with L929 mouse fibroblast cells to estimate cytotoxicity of the hydrogels. Cell behaviors on/in G-quadruplex hydrogels were then examined.

## 2. Results and Discussion

**Figure 2:**
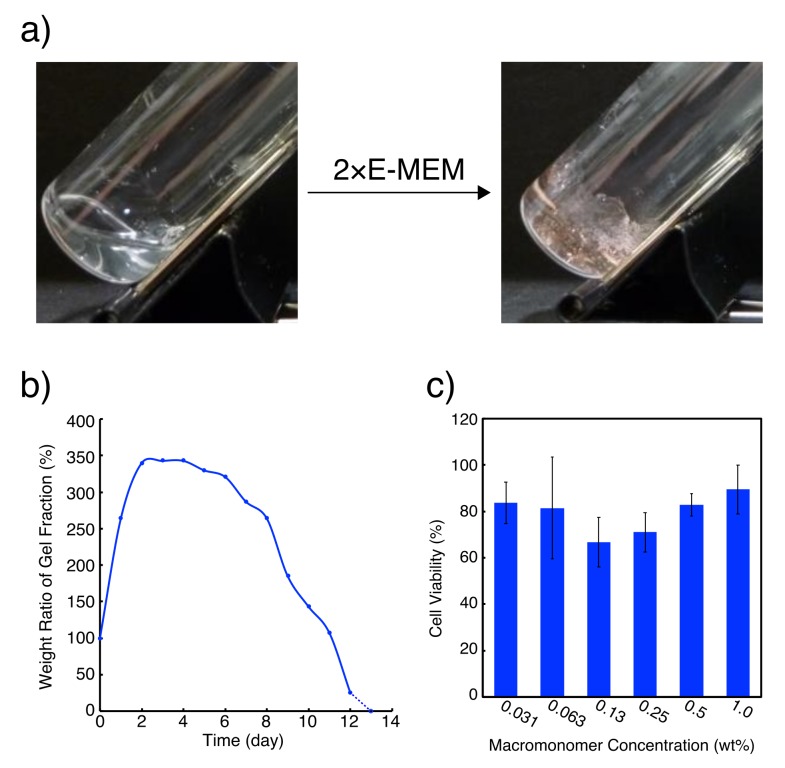
(**a**) Photographs of 20 wt % L4.6k-dG4 before (**left**) and after (**right**) the addition of equal volume of 2×E-MEM. (**b**) Swelling and erosion profile of DNA quadruplex hydrogels in 1×E-MEM at 37 °C. (**c**) WST-8 cell viability assay of L929 mouse fibroblast cells treated with L4.6k-dG4 solution. Cells were cultured in 96-well plates in the presence of L4.6k-dG4 at various concentrations for 24 h. Cells without L4.6k-dG4 treatment were used as negative control. Cell viability was 84 ± 9, 82 ± 22, 67 ± 11, 71 ± 8, 83 ± 5, and 90 ± 11% in 0.031, 0.063, 0.13, 0.25, 0.50, and 1.0 wt % L4.6k-dG4 solution, respectively.

We chose L4.6k-dG4 [25], in which four dG residues were coupled to both of the ends of linear PEG4.6k with modified HELP synthesis (Figure 1b), as the macromonomer to prepare G-quadruplex hydrogels. When equal volume of 2×Eagle′s minimal essential medium (E-MEM) was added to 30 wt % L4.6k-dG4 stock solution, quite stiff hydrogel, which was still stable even at 37 °C, was obtained (Figure 2a). Concentration of Na^+^ in 1×E-MEM is ca. 120 mM and it is far above the critical gelation concentration for Na^+^, which is ca. 20 mM for 15 wt % L4.6k-dG4 hydrogel [25]. The resulting hydrogel was also stable under continuous exposure to cell culture media (Figure 2b). When double the volume of 1×E-MEM was added on to 15 wt % L4.6k-dG4 hydrogel, the gel swelled up to 350% weight ratio within 2 days; kept the weight for about 5 days, then almost linearly lost weight in the next 7 days.

We then examined cytotoxicity of the PEG-ODN conjugate (Figure 2c). Aqueous L4.6k-dG4 solution was diluted from 1 wt % with equal volume of 1×E-MEM (containing FBS) in five stages down to 0.031 wt % and was added to L929 fibroblast culture. Cell viability after 24 h in the presence of L4.6k-dG4, estimated with WST-8 assays, was all within 60–90% without significant difference for any concentration. Even addition of 1 wt % L4.6k-dG4 solution resulted in 90% viability, showing that cytotoxicity of L4.6k-dG4 is almost negligible.

**Figure 3:**
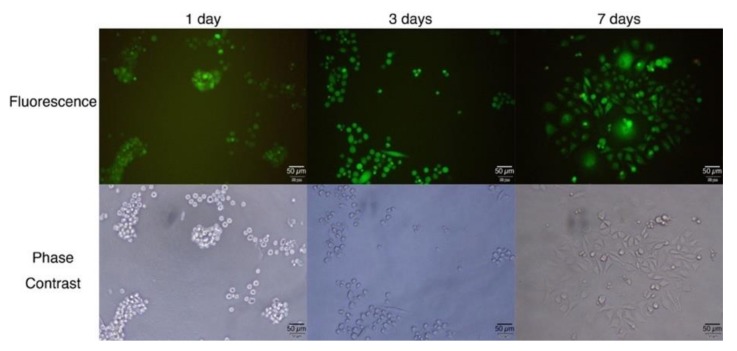
Fluorescence microscope images of cultured L929 fibroblast cells deposited on the surface of DNA quadruplex hydrogels. Cells with green fluorescence are alive, whereas dead cells are stained in red.

Next, L929 fibroblast cells were applied to and cultured on G-quadruplex hydrogels (Figure 3). Cells were applied to the surface of 15 wt % G-quadruplex hydrogel containing 1×E-MEM and incubated for a predetermined time before being treated with LIVE/DEAD^®^ assay kit. Even after 1 day of incubation, most of the cells stayed spherical and tended to aggregate with each other. The aggregates became larger over time, though most of the cells were still alive. Some (ca. 7%) extended cells were observed after 3 days. After 7 days, the majority (ca. 70%) of the cells were extended ones on the bottom of the well. Cells probably sedimented gradually through the hydrogel keeping their spherical shape, as the 3D network of PEG in G-quadruplex hydrogel can be slowly rearranged due to the reversible nature of G-quadruplexes, and finally adhered to the glass substrate in the bottom to start proliferation.

**Figure 4:**
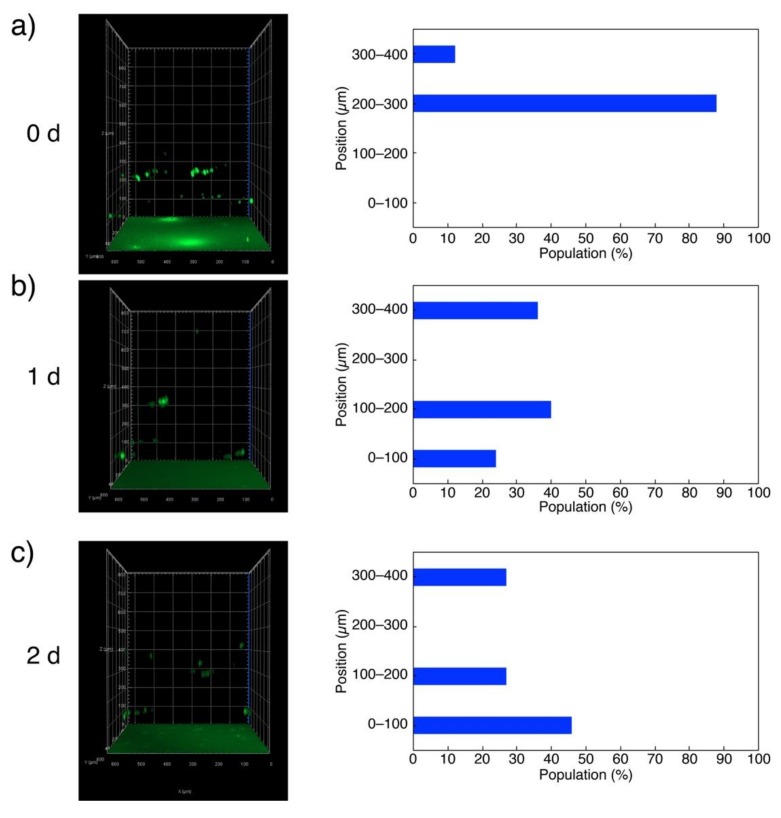
Confocal laser scanning fluorescence microscope (CLSM) 3D images of L929 fibroblast cells labeled with CellTracker™ Green dye and deposited on 15 wt % L4.6k-dG4 hydrogels after 0 day (**a**), 1 day (**b**), and 2 days (**c**).

The above argument was further confirmed by z-stack 3D analysis of the hydrogel using a CLSM (Figure 4). The cells labeled with CellTracker™ Green dye and deposited on 15 wt % G-quadruplex hydrogel were initially found within 200–300 nm (12% population) and 300–400 nm (88%) zones from the bottom (Figure 4a). The majority of the cells then moved to 100–200 nm (40%) and 0–100 nm (24%) zones in 1 day, while those that stayed in 300–400 nm were ca. 36% of the total population (Figure 4b). After 2 days, almost half of the cells (46%) reached the bottom of the well, whereas the populations in 300–400 and 100–200 nm zones were both 27% (Figure 4c).

## 3. Conclusions

It is shown that Na^+^-responsive G-quadruplex hydrogels are not harmful for living cells, but, just like in many other PEG-based hydrogels [30,31,32,33,34,35,36,37], the cells cultured in G-quadruplex hydrogels stay spherical and do not proliferate since they cannot adhere to the matrix. The addition of other substrates that allow cell adhesion such as collagen to hydrogel matrix may solve this problem and promote cell proliferation [36,37]. The present system with high biocompatibility and biodegradability might provide efficient cell storage media that does not require freezing as well—if the observed sedimentation of the cells can be prevented, for example, by adjusting the specific gravity of G-quadruplex hydrogels [38,39,40].

## 4. Materials and Methods

### 4.1. Materials

PEG-ODN conjugate used in this study (L4.6k-dG4) was synthesized with modified-HELP according to the method described in reference [25]. Eagle′s minimal essential medium (E-MEM), 10×phosphate buffered saline (PBS(-)), and 1×PBS(-) were purchased from Wako Pure Chemical (Osaka, Japan). Fetal bovine serum (FBS) was purchased from BioWest (Nuaillé, France). WST-8 Cell Proliferation Assay Kit was purchased from Dojindo Laboratories (Kumamoto, Japan). LIVE/DEAD^®^ Viability/Cytotoxicity Kit and CellTracker™ Green CMFDA dye were purchased from Thermo Fisher Scientific Inc. (Whaltham, MA, USA). L929 mouse fibroblast cells were obtained from ECACC.

### 4.2. Preparation of G-Quadruplex Hydrogel Involving 1×E-MEM

For preparation of 15 wt % L4.6k-dG4 hydrogel, 35.3 mg of L4.6k-dG4 was first dissolved in 100 µL of milliQ water in a glass test tube to give 30 wt % stock solution, then 100 µL of 2×E-MEM was added dropwise to the mixture.

### 4.3. Exposure of G-Quadruplex Hydrogel to Cell Culture Media

An amount of 400 µL of 1×E-MEM was added onto 15 wt % L4.6k-dG4 hydrogel prepared as above and the system was incubated at 37 °C. After predetermined time, the supernatant was collected and the remaining hydrogel was weighed. Collected supernatant was then returned to the system for further measurements.

### 4.4. Cytotoxicity Estimation of PEG-ODN Conjugate

L929 mouse fibroblast cells (5 cells/µL in 100 µL 1×E-MEM (containing FBS)) were first cultured in 96-well plates at 37 °C for 24 h and then the supernatant was removed. Aqueous L4.6k-dG4 conjugate solution was diluted from 1 wt % with equal volume of 1×E-MEM (containing FBS) in 5 stages and added to each well. After 21 h, 10 µL of WST-8 mixture was added; the absorbance at 450 nm was measured 3 h later by using a microplate reader (BIO-RAD iMark^TM^ Microplate Reader).

### 4.5. Culture of Cells Deposited on G-Quadruplex Hydrogel Surface

In 96-well plates, 15 wt % L4.6k-dG4 hydrogels were prepared. First, 30 wt % L4.6k-dG4 stock solution was prepared as described above. Then, 35 μL of the stock solution and 35 μL of 2×E-MEM were added to each well and mixed thoroughly by pipetting until gelation. L929 fibroblast cell suspension (50 cells/µL, 100 µL) was slowly loaded onto the hydrogels and incubated for a predetermined time. After the supernatant was removed, the hydrogels were washed once with 1×PBS(-). Then, 100 μL of LIVE/DEAD^®^ assay solution, containing calcein AM and EthD-1, was added to each well and the plate was incubated for another 2 h. Fluorescence from the cells (green for calcein representing living cells and red for EthD-1 for dead cells) was observed with a confocal laser scanning microscope (Zeiss LSM 800). For CLSM z-stack 3D analysis, cells were labeled with CellTracker™ Green dye according to the instruction manual provided in the kit before application to the wells.
